# From Glacial Refugia to Future Shifts: Unraveling the Spatiotemporal Dynamics of Endangered *Acer sutchuenense* Franch. Under Climate Change

**DOI:** 10.3390/biology15050397

**Published:** 2026-02-28

**Authors:** Xinhe Xia, Xianjun Yang, Sanyao Li, Wujun Xiang, Lixia He, Zhongqin Luo

**Affiliations:** 1 College of Agriculture and Forestry Ecology, Shaoyang University, Shaoyang 422000, China; xinhex355@163.com (X.X.); yxj0008@163.com (X.Y.); helixia0605@163.com (L.H.); 2 Hunan Provincial Key Laboratory of Germplasm Innovation and Ecological Cultivation of Regionally Characteristic Native Plants in the Nanshan National Park Region, Shaoyang University, Shaoyang 422000, China; 3 Shaoyang City Forestry Bureau, Shaoyang 422000, China; lisanyao1107@126.com; 4 Tangtianshi Town Agricultural Comprehensive Service Center, Shaoyang 422000, China; jacksonxiang@163.com

**Keywords:** climate change, *Acer sutchuenense*, potential habitat, MaxEnt, glacial refugia

## Abstract

Given that *Acer sutchuenense* Franch., an endangered maple endemic to China, severely threatened by habitat degradation and climate fluctuations, understanding its spatiotemporal dynamics is crucial for formulating conservation strategies. Herein, climatic, topographic and soil variables were employed to simulate historical, present, and future distribution patterns of *A. sutchuenense* using the optimized MaxEnt model. Our results indicated that Mean Temperature of Driest Quarter (Bio9) and Temperature Seasonality (Bio4) were the key environmental drivers. Since the Last Interglacial, *A. sutchuenense* had experienced a continuously reduction in its suitable area, though the mountains surrounding the Sichuan Basin functioned as vital glacial shelters. Although the potential suitable habitat was distributed in a ring shape, *A. sutchuenense* occurs only on the east and west sides of the Sichuan Basin, probably due to the terrain complexity and limited dispersal ability. In the future, *A. sutchuenense* faces a westward contraction and a migration lag behind climate velocity due to dispersal constraints. Overall, we recommend a multi-dimensional conservation framework that prioritizes in situ conservation in core refugia, urgently establishes ecological corridors to facilitate eastward migration under climate change, implements ex situ conservation through germplasm collection for vulnerable southwestern populations, and enhances long-term monitoring to ensure species persistence.

## Simple Summary

*Acer sutchuenense* is a rare and endangered maple endemic to China, facing severe threats due to habitat loss and rapid climate change. In this study, a MaxEnt model optimized using the kuenm package in R 4.3.1 software was employed to simulate its spatiotemporal distribution. Although the mountains surrounding the Sichuan Basin provided glacial refugia for this species during ancient climate changes, its distribution has now split into two separate regions. This split is likely due to the rugged terrain and the limited natural seed dispersal ability of this species over long distances. Due to the projected migration lag, the natural migration of *A. sutchuenense* may fail to keep up with the pace of future climate warming. This could lead to the loss of nearly half of its unique genetic resources, especially in its western habitat. Therefore, implementing a framework that combines in situ and ex situ conservation, and establishing ecological corridors, is crucial for the long-term survival and genetic integrity of *A. sutchuenense*.

## 1. Introduction

Climate change may be a primary driver of habitat loss and fragmentation, increasing the risk of species extinction, especially for endangered species [1,2]. The repeated and intense climate oscillation during the Quaternary Ice Age and interglacial period led to most parts of Europe, North America and northern Asia being repeatedly in the process of glacial advance and retreat [3,4]. In order to adapt to these environmental shifts, species were compelled to migrate or undergo adaptive evolution to secure suitable habitats [5,6]. Although China experienced relatively less direct impact from Quaternary glaciation compared to Europe, climatic fluctuations still acted as a key driver for migration and adaptive evolution of numerous species [3,4].

Global climate change, characterized by increasing extreme climate events and a projected temperature rise [7], poses a significant threat to endangered plants [8,9]. It is well established that climate warming is a primary driver of shifts in species distributions [10,11,12]. A representative example is the prediction by Jay et al. (2012), which estimated that a temperature rise of 2 °C and 4 °C would displace the distribution boundaries of alpine plants northeastward by approximately 92 km and 188 km, respectively [13]. As a result, species distribution models (SDMs) are increasingly used to evaluate how future climate shifts may alter the suitable ranges for plants, especially for endangered plants [14,15]. However, current research primarily focuses on species’ suitable habitats under present and future climates [16,17,18], often neglecting key constraints such as historical climate variability and dispersal capacity, which limits reliable predictions of future migration.

Belonging to the Sapindaceae family, the genus *Acer* L. includes about 200 species [19] and offers not only significant ornamental value but also diverse utilities such as medicinal [20] and timber values [21]. *Acer sutchuenense* Franch., an endangered maple endemic to China, is sparsely distributed in the mountainous regions of Sichuan, northwestern Hunan, and western Hubei at elevations ranging from 1000 to 2500 m [22,23]. As one of only nine compound-leaved maple species among the more than 200 in the genus *Acer*, it is a key species for elucidating the origin, evolution, and mechanisms of endangerment within the genus [24]. However, the species is facing the dual threats of urban expansion and habitat degradation, precipitating a sharp decline in its wild populations, which is now only distributed in isolated areas on the east and west sides of the Sichuan Basin [23]. Nevertheless, in most of the existing literature, it has only been briefly mentioned within the framework of plant resource surveys or phylogenetic studies of the *Acer* genus [24,25], lacking in-depth discussions on its general ecological requirements and its climatic responses.

We hypothesize that the current fragmented distribution of *A. sutchuenense* was primarily shaped by Quaternary glacial–interglacial cycles. Given the dual pressures of ongoing climate and habitat change, it is crucial to understand both the historical drivers behind this distribution pattern, the species’ dispersal capabilities, and the likely response to future climate shifts to inform conservation strategies. Therefore, by integrating data from field surveys and historical records, this study aimed to (i) identify the key environmental drivers influencing *A. sutchuenense*; (ii) reconstruct its historical dynamics to unravel the mechanisms underlying its current geographical distribution; and (iii) simulate its future suitable distribution areas to inform the long-term genetic resources conservation and management strategies.

## 2. Materials and Methods

### 2.1. Species Distribution Records

**Figure 1:**
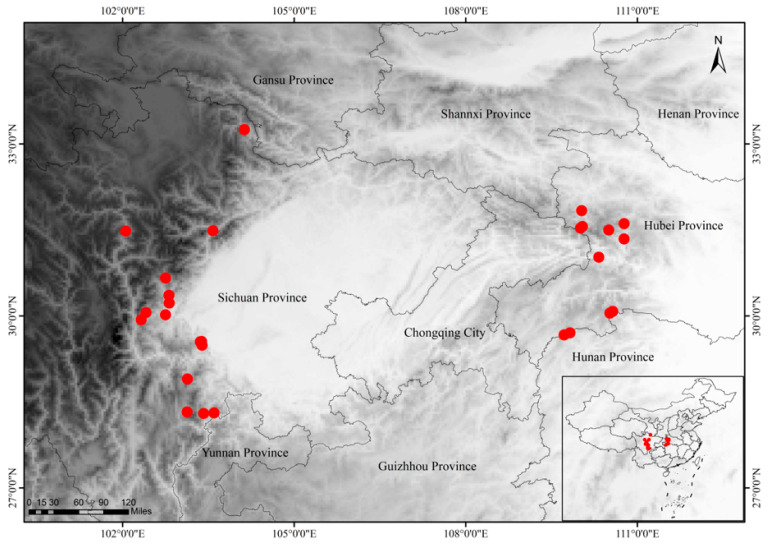
Occurrence records of *A. sutchuenense* used to construct MaxEnt models. Note: Red bullets represent the distribution points of *A. sutchuenense*.

42 occurrence records of *A. sutchuenense* were compiled from field surveys, literature, and herbarium databases, including the Global Biodiversity Information Facility (http://www.gbif.org/search?q=Acer sutchuenense) (accessed on 14 September 2025) and the Chinese Virtual Herbarium (http://www.cvh.ac.cn/) (accessed on 14 September 2025). To reduce spatial sampling bias and enhance model reliability, the occurrence records were spatially thinned to a minimum distance of 2.5 km using ENMTools 1.0 [26]. Ultimately, a final set of 27 spatially independent occurrence records were used for subsequent analysis (Figure 1).

### 2.2. Environmental Factors Sourcing and Screening

19 climate factors, three topographic factors and 15 soil factors (Table S1) were used to simulate the potential suitable areas over different periods: the past (Last Interglacial (LIG), Last Glacial Maximum (LGM), Mid–Holocene (MH)), present (1970–2000), and future (2041–2061 and 2081–2100). Climate and elevation factors were required from the WorldClim data website (http://worldclim.org) (accessed on 19 September 2025) at a 2.5 arc-minutes resolution. Slope and aspect were derived from elevation using ArcMap 10.7. Soil factors originated from the Harmonized World Soil Database at 30 arc-second resolution (https://www.fao.org/soils-portal/en/) (accessed on 8 October 2025) [27]. To maintain spatial uniformity, soil factors were resampled to a 2.5 arc-minutes resolution.

Correlation analysis of the 37 environmental drivers was performed using ENMTools 1.0 (Figure S1). Variables with contribute rate ≤0.1% were excluded; subsequently, for highly correlated pairs (|r| > 0.80), only the predictor with higher contribution was retained to mitigate multicollinearity [28,29]. Finally, a total of 11 variables were selected (Table 1).

### 2.3. Model Establishment and Optimization

Model calibration was performed using the kuenm package of R 4.3.1 [30] to optimize the feature class (FC) and regularization multiplier (RM). 75% of distribution records were selected as the training set and 25% as the test set. RM was assigned a range of 0.1–4.0, increasing by 0.1 in turn. Among the 1240 candidate combinations, the optimal model was identified based on an omission rate < 5% and the minimum AICc value [27,31].

The receiver operating characteristic (ROC) curve was used to evaluate the model performance. The area under the curve (AUC) ranges from 0 to 1, with higher value indicating superior predictive accuracy. Generally, an AUC score greater than 0.9 generally indicates excellent predictive ability [32,33]. Taking the occurrence probability > 0.5 as the threshold, the suitable ranges and peak values of selected variables were analyzed.

### 2.4. Prediction of Potential Suitable Habitats for A. sutchuenense

MaxEnt 3.3 [34] was employed to analyze the potential suitable habitats of *A. sutchuenense* with 10 replicates and the mean value was adopted as the final output. Habitat suitability was classified into four levels using ArcMap 10.7: unsuitable (0–0.1), low suitability (0.1–0.3), moderate suitability (0.3–0.6), and high suitability (0.6–1.0).

To further quantify the fluctuations in suitable area and the trends in centroid migration for *A. sutchuenense* across different periods, habitat areas were calculated in ArcMap 10.7 based on pixel size. Additionally, the SDMToolbox 2.4 was employed to visualize the spatio-temporal dynamics of suitable habitats, and to map the migration trajectories of the highly distributional centroids [35].

## 3. Results

### 3.1. Model Performance Optimization and Assessment

From the 1240 model combinations, the configuration with FC of Q + T and RM of 2 achieved an omission rate below 5% and a delta_AICc value of zero, indicating that an optimal balance has been reached between model complexity and fit. The model demonstrated outstanding performance under current conditions, with the AUC value of 0.988 ± 0.003, indicating its high accuracy and reliability (Figure S2).

### 3.2. Key Environmental Drivers

**Figure 2:**
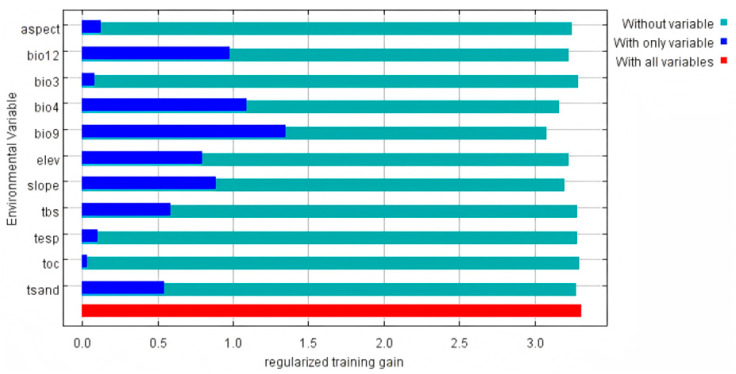
Jacknife test of 11 environment variables in MaxEnt.

Bio9 and Bio4 had the highest permutation importance, with a combined permutation importance of 87.7% (Table 1). In addition, the jackknife test showed that Bio9, Bio4, Bio12, Slope and Elevation had high gain values (>0.8), with Bio9 being the highest (Figure 2). It was indicated that temperature was the predominant variable of the potential distribution for *A. sutchuenense*, exceeding the influence of precipitation, topography, and soil factors.

Taking a threshold of >0.5, the optimal environmental conditions for *A. sutchuenense* were identified as follows: Bio9, −1.46~5.94 °C (peak: 1.59 °C); Bio4, 551.49~798.85 (peak: 642.07); Bio12, 718.60~1690.57 mm (peak: 1227.73 mm); Slope, >1.8°, and Elevation, 1236.28~3044.63 m (peak: 2920.98 m) (Table 2, Figure S3).

### 3.3. Suitable Distribution During Present Climate Scenarios

**Figure 3:**
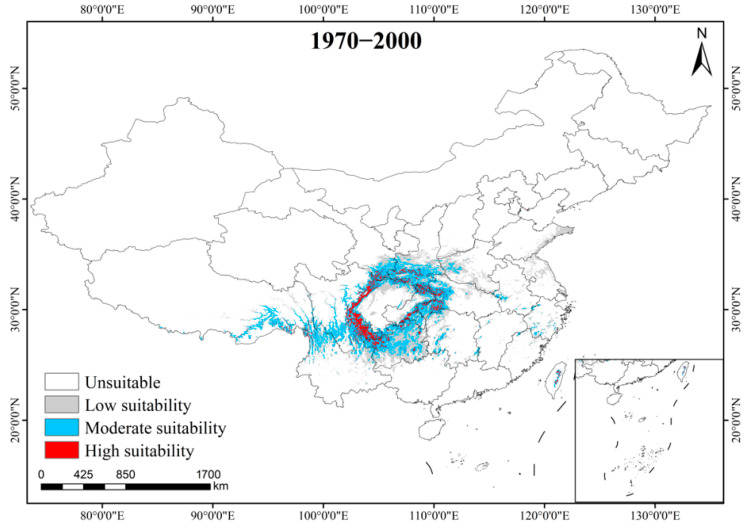
Distribution of potential suitable habitats for *A. sutchuenense* in the current period.

The present total suitable habitat area for *A. sutchuenense* was 87.94 × 10^4^ km^2^, with areas of high, moderate, and low suitability accounting for 7.68, 31.64, and 48.62 × 10^4^ km^2^, respectively. The highly suitable regions were mainly concentrated around the Sichuan Basin, including central Sichuan, central Chongqing, western Hubei, northeastern Yunnan, northwestern Guizhou, and southern Shaanxi, as well as a few scattered in the central Taiwan and southeastern Xizang. Moderately suitable areas extend outward from the edges of the highly suitable areas, while lowly suitable areas were mainly distributed in Guizhou, eastern Henan, central Shaanxi, and eastern Shandong (Figure 3). Compared with its potential distribution range predicted by the model, the actual range of *A. sutchuenense* showed a distinct east–west disjunction around the Sichuan Basin, with many potential highly suitable areas remaining without distribution.

### 3.4. Spatiotemporal Dynamics of Potential Suitable Habitats During Historical Climate Scenarios

**Figure 4:**
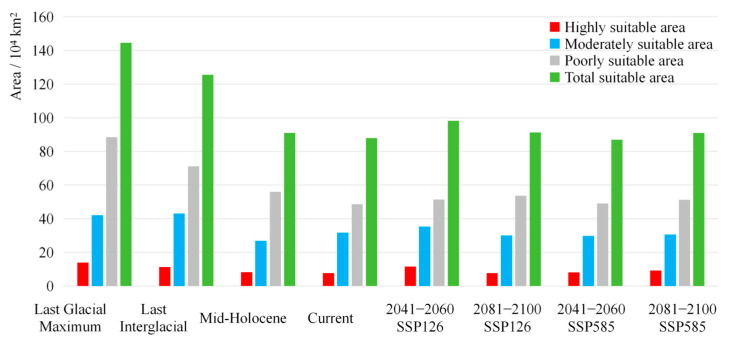
Dynamic changes in the potential suitable habitat of *A. sutchuenense* in different periods.

The suitable habitat of *A. sutchuenense* had been continuously shrinking in the past, mainly due to the destruction of the habitats in the Sichuan Basin and the southeastern China. The maximum distribution occurred during LIG, with a total suitable area of 144.61 × 10^4^ km^2^ (High: 13.95 × 10^4^ km^2^; Moderate: 42.14 × 10^4^ km^2^; Low: 88.52 × 10^4^ km^2^). Subsequently, there was a reduction during LGM, where the total and highly suitable areas decreased to 125.51 × 10^4^ km^2^ and 11.21 × 10^4^ km^2^, respectively. By the MH, these figures further dropped to 91.00 × 10^4^ km^2^ and 8.17 × 10^4^ km^2^ (Table 3, Figure 4). This might be because the alternating periods of cooling and warming during ice ages and interglacial periods gradually made the suitable growth range of *A. sutchuenense* increasingly narrower.

**Figure 5:**
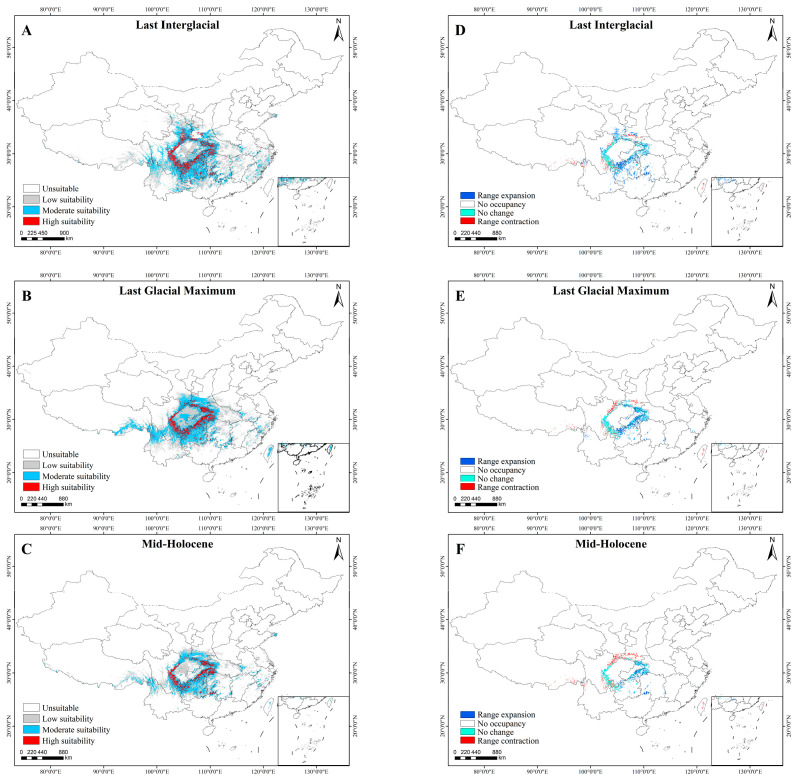
Potential historical distribution (**A**–**C**) and range shifts of highly suitable habitats (**D**–**F**) for *A. sutchuenense*. (**A**,**D**) Last Interglacial. (**B**,**E**) Last Glacial Maximum. (**C**,**F**) Mid-Holocene.

Compared to the present period, the highly suitable areas for *A. sutchuenense* during the LIG mainly contracted westward but expanded eastward, southward, and northward. During the LGM and MH, the highly suitable areas expanded towards the southeast, while a contraction occurred in the northwest of its distribution range (Figure 5). In summary, the historical distribution dynamic of *A. sutchuenense* mainly showed a trend of westward contraction and eastward expansion.

### 3.5. Spatiotemporal Dynamics of Potentially Suitable Areas Under Future Climate Scenarios

Under SSP126 (Sustainable Development Pathway), the total suitable area of *A. sutchuenense* was projected to initially expand and then decline. However, compared to the current period, its total suitable area was still showing an overall expanding trend. In contrast, the highly suitable area was also projected to show an initial increase followed by a decrease, leading to an overall contraction. Specifically, the total and highly suitable areas were projected to reach 98.24 × 10^4^ km^2^ and 11.58 × 10^4^ km^2^ during 2041–2060, but were expected to contract to 91.30 × 10^4^ km^2^ and 7.66 × 10^4^ km^2^ by 2081–2100. Compared to the current period, the highly suitable area for *A. sutchuenense* was projected to expand outward in all directions during 2041–2060. However, expansion was projected in the east, while extensive contraction was projected in other directions during 2081–2100 (Table 3, Figure 4).

Under SSP585 (conventional development path), the total suitable area of *A. sutchuenense* was projected to initially decline and then expand, leading to an overall upward trend. However, the highly suitable area was expected to show a continuous increase. During the 2041–2060 period, the total suitable area was projected to decrease to 86.93 × 10^4^ km^2^, while the highly suitable area was expected to increase to 8.11 × 10^4^ km^2^. By 2081–2100, the total and highly suitable areas were projected to reach 90.05 × 10^4^ km^2^ and 9.10 × 10^4^ km^2^, respectively. Compared to the current period, the highly suitable area will expand towards the north, but contract significantly in the south during 2041–2060. From 2081 to 2100, the highly suitable habitat was projected to shift towards the northeast, while contracting in the southwest (Table 3, Figure 4).

**Figure 6:**
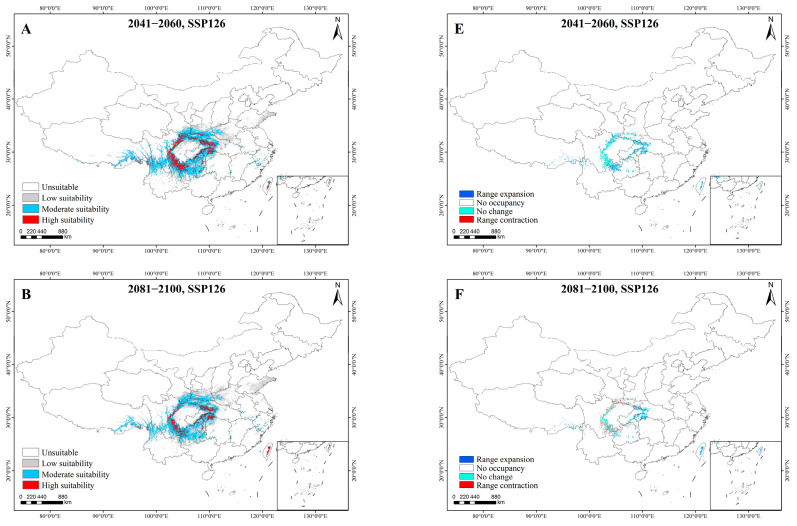

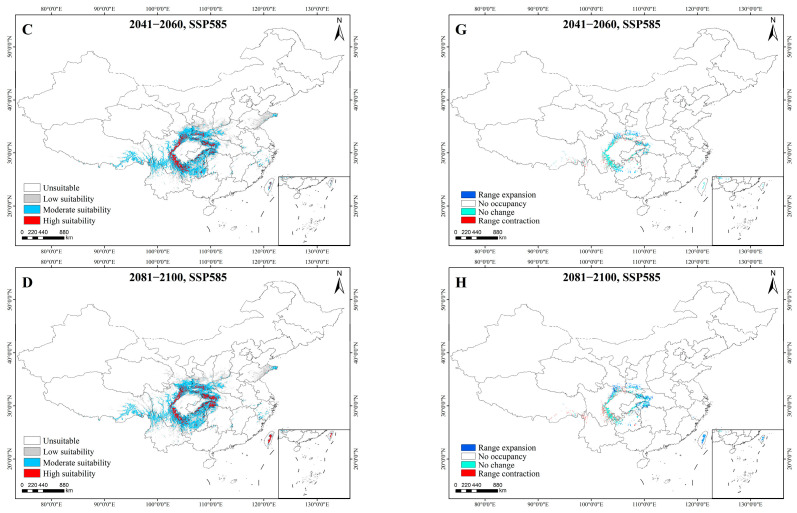
Potential future distribution (**A**–**D**) and range shifts of highly suitable habitats (**E**–**H**) for *A. sutchuenense*. (**A**,**E**) 2041–2060, SSP126. (**B**,**F**) 2081–2100, SSP126. (**C**,**G**) 2041–2060, SSP585. (**D**,**H**) 2081–2100, SSP585.

In summary, the potential total and highly suitable areas of *A. sutchuenense* were projected to remain relatively stable in the future, showing an overall upward trend compared to the current period. Additionally, the highly suitable area of *A. sutchuenense* was projected to mainly shift eastward, while contracting in the west (Figure 6), which was consistent with its historical distribution pattern.

### 3.6. Centroid Shifts in the Highly Suitable Habitat of A. sutchuenense

The centroid of the historical highly suitable area of *A. sutchuenense* initially moved eastward, and then shifted westward. During the LIG, the centroid was located in Jiulongpo District, Chongqing (106.34° E, 29.52° N). Over time, it moved 31.27 km northeast to Yubei District (106.62° E, 29.66° N) during the LGM. By the MH, the centroid had moved 25.40 km southeast to Banan District (106.87° E, 29.61° N), and subsequently shifted 131.33 km northwest to Anyue County, Sichuan Province (105.58° E, 29.96° N), forming the current distribution pattern.

**Figure 7:**
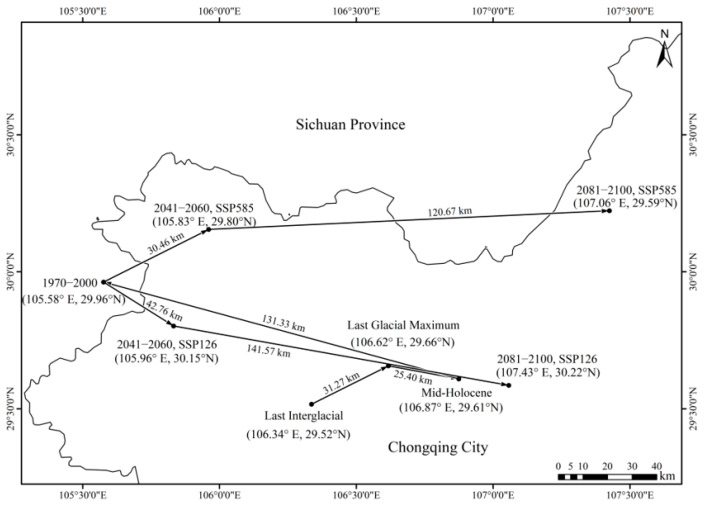
Shifting centroids of the highly suitable area based on different climatic scenarios.

In the future, the centroid of highly suitable habitat for *A. sutchuenense* was projected to move predominantly eastward, with a southeastward migration under SSP126 and a northeastward migration under SSP585. Under SSP126, the centroid was projected to migrate 30.46 km southeast to Tongliang District, Chongqing (105.83° E, 29.80° N) during 2041–2060, and to continue southeast by 120.67 km to Dianjiang District (107.06° E, 29.59° N) by 2081–2100. Conversely, under SSP585, the centroid was projected to shift 42.76 km northeast to Tongliang District (105.96° E, 30.15° N) in 2041–2060, and to further migrate 141.57 km northeast to Fuling District (107.43° E, 30.22° N) by 2081–2100 (Figure 7).

## 4. Discussion

### 4.1. Influences of Key Environmental Drivers on the Distribution of A. sutchuenense

Permutation importance was used to identify the primary environmental drivers of *A. sutchuenense* distribution, as it depends exclusively on the final model and offers a more robust assessment of variable significance [36,37]. Herein, Bio9 and Bio4 exhibited the highest permutation importance for *A. sutchuenense*. This pattern was consistent with findings for many other subtropical plants, where temperature-related factors often serve as primary distribution constraints [38,39]. Similarly, the northern range boundaries of organisms across the Northern Hemisphere are primarily shaped by seasonal temperature fluctuations and winter cold stress [40,41]. Bio9 reflects the low-temperature stress faced by *A. sutchuenense* when water resources are scarce. If the Bio9 value is too low, it may cause the vulnerable seedlings and dormant buds to suffer frost damage [42]. On the other hand, if the Bio9 value is too high, it may interfere with the plants’ ability to meet the required low-temperature conditions for breaking dormancy, thereby having an adverse effect on the spring phenology and germination [43,44]. Meanwhile, Bio4 is used to measure the seasonal changes in temperature. Excessively low Bio4, such as in tropical regions, fails to provide the necessary temperature cues to trigger leaf abscission or sprouting in *A. sutchuenense*. Conversely, excessively high Bio4 indicates extreme thermal fluctuations that likely surpass the species’ physiological tolerance limits. Thus, Bio9 and Bio4 collectively constrain the latitudinal and altitudinal limits of *A. sutchuenense*, restricting it to specific mountainous area within the subtropical zone that experience cool but not severely cold winters.

Furthermore, water availability serves as a critical determinant of plant distribution patterns [45]. As the dominant hydrological driver, Bio12 contributed 26.5% to the model, with an optimal range of 718.60~1690.57 mm for the distribution of *A. sutchuenense*. Previous studies have also identified similar patterns [40,46]. Bio12 has defined its drought threshold of distribution, and the water limitation in the high-altitude areas of western China constitutes a significant environmental filtering effect [47,48], which becomes a key factor hindering the expansion of this species to the west.

Apart from climatic factors, the geographical distribution of *A. sutchuenense* was also influenced by the combined effects of topography and soil factors. Intensified soil surface warming and evaporation under future climate scenarios will further heighten soil water loss and drought stress [49]. Acting as a primary vertical filtering driver, altitude determines the vertical distribution of temperature and precipitation [50]. Within a specific altitude zone, slope regulates drainage, light, and soil layer thickness, further shaping the micro-habitats [51,52]. At the same time, the Topsoil Base Saturation (T_BS) not only ensures the availability of essential cations (e.g., Ca^2+^, Mg^2+^), but also enhances soil acid resistance [53]. In summary, the interplay of these factors had shaped the unique ecological niche of *A. sutchuenense*.

### 4.2. Historical Spatiotemporal Dynamics and Potential Glacial Refugia of A. sutchuenense

Since LIG, the potential highly suitable habitat of *A. sutchuenense* had undergone continuous contraction and complex spatiotemporal evolution. During LGM and MH, a sharp drop in global temperatures or an imbalance in the distribution of water and heat led to the continuous retreat of the northern distribution limit of this species. Throughout its long evolutionary history, mountainous areas with diverse terrain and micro-environments often served as glacial refugia [54,55]. In these regions, plants adapted to the continuously declining temperatures by migrating to lower altitudes within the mountains, and then expanding back to higher altitude areas as temperatures rose [56,57]. Chen et al. (2011) reviewed the impact of the Quaternary glaciers in China, pointing that the Hengduan Mountains, Central China, and the Nanling Mountains might have been potential continental refugia that preserved rich biodiversity [6]. It is notable that the Hengduan Mountains and Central China are considered the origin and diversification centers of the *Acer* genus [58]. The Hengduan Mountains alone harbor 58 *Acer* species, accounting for approximately 41.4% of the total in China and 29% of the global total [59]. Interestingly, despite the drastic environmental fluctuations during LGM, our results indicated that the mountainous areas surrounding the Sichuan Basin (such as the Daba Mountains, Shennongjia, and the Qionglai Mountains within the Hengduan range) maintained a relatively high habitat suitability. This finding supports the hypothesis that the mountains in central and southwestern China were once important plant refugia in East Asia [6,60]. The complex terrain of these regions effectively blocked the cold air from the north, providing stable micro-environments for *A. sutchuenense*, allowing it to survive during the ice age.

Under current climate scenarios, although the potential highly suitable area shows a clear northward expansion trend, the natural populations of *A. sutchuenense* have not been recorded in the northern regions within the suitable range (such as southern Gansu and Shaanxi). This situation is also frequently observed in other species of the *Acer* genus [61], and it may be due to the limitations of seed dispersal, which prevent long-distance migration [62]. Currently, the actual distribution of *A. sutchuenense* is isolated between the eastern and western sides of the Sichuan Basin. Future research could integrate population genomics to explore the genetic differentiation and evolutionary history of these two isolated geographic groups, which would lay a scientific foundation for the conservation of *A. sutchuenense*.

### 4.3. Future Climate Responses and Conservation Priorities for A. sutchuenense

Our findings indicated that the potential distribution of *A. sutchuenense* would expand in the future, suggesting its considerable adaptive potential to global warming. This expansion is likely driven by the optimization of hydrothermal conditions. Specifically, winter warming reduces the risk of frost damage, while increased summer rainfall alleviates drought stress, jointly ensuring the hydrothermal requirements for the plant’s vegetative growth [63,64]. Furthermore, compared to evergreen conifers, deciduous broad-leaved species are expected to possess a greater competitive advantage under future climate change [65]. Moreover, this positive trend may also be related to the restricted distribution range of *A. sutchuenense*. Previous research on *Rhododendron* has similarly found that species with narrow ranges may respond positively to future climate and land-use changes [66].

However, the expansion in suitable habitat does not necessarily translate to a mitigation of extinction risk. Future projections for the highly suitable areas of *A. sutchuenense* primarily exhibit a westward contraction alongside an northeast/southeast expansion, a trend largely consistent with the findings of Jay (2012) [13]. Although this spatial shift expands the potential suitable range, it poses a significant challenge to the natural dispersal ability of *A. sutchuenense*. Given the rugged terrain and severe anthropogenic disturbances in its distribution area, the actual migration may lag behind the pace of climate change [67]. Moreover, climate change may also disrupt the reproductive ecology of *A*. *sutchuenense*. Since most species of the *Acer* genus are insect-pollinated [68,69], climate change may cause the phenological periods of the plants and their pollinators to mismatch. This asynchrony may undermine reproductive success, hinder seed germination and increase the mortality rate of seedlings, thereby creating additional obstacles for the natural spread of the species [70]. The consequences of this lagging migration speed include not only the disappearance of local populations that fail to keep up, but also the loss of their unique genetic resources. The current discontinuity in distribution between the east and west indicates a long-term phenomenon of population isolation, which may have led to distinct genetic adaptations in the western and eastern groups. It is expected that the shrinking of the distribution range in the west will result in the disappearance of local adaptive alleles, which are crucial for the evolution and adaptability of the species [71]. Therefore, it is necessary to take urgent conservation measures for these genetic resources.

In situ and ex situ conservation are two core strategies for protecting endangered plants [72]. In situ conservation can maintain the evolutionary potential of the species, while ex situ conservation serves as an important backup measure to prevent species extinction. Only by combining these two approaches and following comprehensive and representative principles can we maximize the protection of the genetic resources of the species [73]. Therefore, it is recommended to adopt a multi-dimensional protection and management framework to safeguard *A. sutchuenense*. Firstly, a comprehensive field investigation should be conducted to fully understand the distribution of *A. sutchuenense*. Secondly, in situ conservation should be prioritized within core refugia, particularly in stable, highly suitable regions such as central Sichuan and the border between Chongqing and Hubei. On this basis, in response to the trend of the predicted distributional centroid moving significantly eastward, it is urgent to construct ecological corridors between the current distribution area and the potential future habitats to mitigate dispersal barriers caused by habitat fragmentation. Meanwhile, for southwestern edge populations facing large-scale contraction, urgent germplasm collection and ex situ conservation should be carried out. Adhering to the principle of ‘the right tree in the right place’ [74], genetic resources should be preserved in national botanical gardens or germplasm banks through artificial introduction. Finally, dynamic monitoring of both natural and cultivated resources should be strengthened to ensure the persistence of *A. sutchuenense*.

## 5. Conclusions

This study identified the key environmental actors influencing the distribution of the endangered *A. sutchuenense*, and its temporal and spatiotemporal dynamic changes. The high permutation importance of Bio9 and Bio4 highlighted the sensitivity of it to winter cold stress and seasonal fluctuations. Model simulations indicated that during the Quaternary climate fluctuations, *A. sutchuenense* was able to survive in the glacial refugia within the mountains surrounding the Sichuan Basin. The total suitable area of *A. sutchuenense* was projected to expand in the future, while its highly suitable area was projected to contract westward and expand eastward. However, due to its limited dispersal ability, the actual migration speed may not be able to keep up with the pace of climate change. These findings highlight the urgency of implementing targeted conservation strategies, including in situ conservation, ex situ conservation, and constructing ecological corridors to assist migration, to ensure the long-term survival of this species.

## Figures and Tables

**Table 1:** 11 Selected variables used for modeling the potentially suitable habitat of *A. sutchuenense*.

Code	Environmental Variables	Contribution Rate	Permutation Importance
Bio12	Annual Precipitation	26.5	3
Slope	Slope	21.8	2.1
T_BS	Topsoil Base Saturation	13.6	1
Bio9	Mean Temperature of Driest Quarter	11.2	58.5
Elev	Elevation	11.1	3.5
Bio4	Temperature Seasonality	7.2	29.2
Bio3	Isothermality	2.9	1.2
Aspect	Aspect	2.7	0.5
T_SAND	Subsoil Sand Fraction	1.5	0.6
T_ESP	Topsoil Exchangeable Sodium Percentage	1	0.4
T_OC	Topsoil Organic Carbon	0.6	0.2

**Table 2:** Suitable range and optimal value of five dominate environmental variables for *A. sutchuenense*.

Environmental Variables	Suitable Range	Optimal Value
Bio9	−1.46~5.94 °C	1.59 °C
Bio4	551.49~798.85	642.07
Bio12	718.60~1690.57 mm	1227.73 mm
Slope	>1.8°	−
Elevation	1236.28~3044.63 m	2920.98 m

**Table 3:** Areas of different suitable habitats for *A. sutchuenense* in different periods.

Period	Highly Suitable Area (10^4^ km^2^)	Moderately Suitable Area (10^4^ km^2^)	Poorly Suitable Area (10^4^ km^2^)	Total (10^4^ km^2^)
LIG	13.95	42.14	88.52	144.61
LGM	11.21	43.12	71.18	125.51
MID	8.17	26.82	56.01	91.00
Current	7.68	31.64	48.62	87.94
2041–2060 SSP126	11.58	35.30	51.36	98.24
2081–2100 SSP126	7.66	30.03	53.61	91.30
2041–2060 SSP585	8.11	29.77	49.05	86.93
2081–2100 SSP585	9.10	30.54	51.31	90.05

## Data Availability

Data are contained within the article and Supplementary Materials. Further inquiries should be directed to the corresponding author.

## Supplementary Materials

The following supporting information can be downloaded at: https://www.mdpi.com/article/10.3390/biology15050397/s1, Figure S1: Correlation analysis of 37 environmental variables; Figure S2: ROC curve simulated in the suitable habitats of *A. sutchuenense*; Figure S3: Response curve for five dominant climate variables; Table S1: 37 environmental variables collected for the MaxEnt model.

## References

[1] Salmona J., Heller R., Quéméré E., Chikhi L. (2017). Climate change and human colonization triggered habitat loss and fragmentation in Madagascar. Mol. Ecol..

[2] Zhang H.L., Chase J.M., Liao J.B. (2024). Habitat amount modulates biodiversity responses to fragmentation. Nat. Ecol. Evol..

[3] Roberts D.R., Hamann A. (2015). Glacial refugia and modern genetic diversity of 22 western North American tree species. Proc. R. Soc. B-Biol. Sci..

[4] Keppel G., Wardell-Johnson G.W. (2012). Refugia: Keys to climate change management. Glob. Change Biol..

[5] Lumibao C.Y., Hoban S.M., McLachlan J. (2017). Ice ages leave genetic diversity ‘hotspots’ in Europe but not in Eastern North America. Ecol. Lett..

[6] Chen D.M., Kang H.Z., Liu C.J. (2011). An overview on the potential Quaternary glacial refugia of plants in China mainland. Bull. Bot. Res..

[7] Zhou T. (2021). New physical science behind climate change: What does IPCC AR6 tell us?. Innovation.

[8] Abdelaal M., AL-Huqail A.A., Alghanem S.M.S., Alhaithloul H.A.S., Al-Robai S.A., Abeed A.H.A., Dakhil M.A., El-Barougy R.F., Yahia A.A. (2024). Population status, habitat preferences and predictive current and future distributions of three endangered *Silene* species under changing climate. Front. Plant Sci..

[9] Urban M.C. (2015). Accelerating extinction risk from climate change. Science.

[10] Parmesan C., Yohe G. (2003). A globally coherent fingerprint of climate change impacts across natural systems. Nature.

[11] Root T.L., Price J.T., Hall K.R., Schneider S.H., Rosenzweig C., Pounds J.A. (2003). Fingerprints of global warming on wild animals and plants. Nature.

[12] Aitken S.N., Yeaman S., Holliday J.A., Wang T., Curtis-McLane S. (2008). Adaptation, migration or extirpation: Climate change outcomes for tree populations. Evol. Appl..

[13] Jay F., Manel S., Alvarez N., Durand E.Y., Thuiller W., Holderegger R., Taberlet P., François O. (2012). Forecasting changes in population genetic structure of alpine plants in response to global warming. Mol. Ecol..

[14] Qazi A.W., Saqib Z., Zaman-ul-Haq M. (2022). Trends in species distribution modelling in context of rare and endemic plants: A systematic review. Ecol. Process..

[15] Merow C., Smith M.J., Silander J.A. (2013). A practical guide to MaxEnt for modeling species’ distributions: What it does, and why inputs and settings matter. Ecography.

[16] Yang Z.B., Bai Y., Alatalo J.M., Huang Z., Yang F., Pu X.Y., Wang R.B., Yang W., Guo X.Y. (2021). Spatio-temporal variation in potential habitats for rare and endangered plants and habitat conservation based on the maximum entropy model. Sci. Total Environ..

[17] Hosseini N., Mehrabian A., Nasab F.K., Mostafavi H., Ghorbanpour M. (2025). Forecasting climate change effects on the potential distribution of *Zhumeria majdae* as an endangered monotypic endemic species: A maxent modeling approach. BMC Ecol. Evol..

[18] Yang L., Zhu X.F., Song W.Z., Shi X.P., Huang X.T. (2024). Predicting the potential distribution of 12 threatened medicinal plants on the Qinghai-Tibet plateau, with a maximum entropy model. Ecol. Evol..

[19] Chase M.W., Christenhusz M.J.M., Fay M.F., Byng J.W., Judd W.S., Soltis D.E., Sennikov A.N., Mabberley D.J., Soltis P.S., The Angiosperm Phylogeny Group (2016). An update of the angiosperm phylogeny group classification for the orders and families of flowering plants: APG IV. Bot. J. Linn. Soc..

[20] He X., Li D.Z., Tian B. (2021). Diversity in seed oil content and fatty acid composition in *Acer* species with potential as sources of nervonic acid. Plant Divers..

[21] Bi W., Gao Y., Shen J., He C.N., Liu H.B., Peng Y., Zhang C.H., Xiao P.G. (2016). Traditional uses, phytochemistry, and pharmacology of the genus *Acer* (maple): A review. J. Ethnopharmacol..

[22] Wu C.Y., Raven P.H., Hong D.Y. (2008). Flora of China: Oxalidaceae Through Aceraceae.

[23] Crowley D. (2020). *Acer sutchuenense*. The IUCN Red List of Threatened Species 2020: E.T193876A2288054. https://www.iucnredlist.org/species/193876/2288054.

[24] Xia X.H., Yu X.D., Fu Q.D., Zhao Y.X., Zheng Y.Q., Wu Y.X., Zhang C.H. (2022). Comparison of chloroplast genomes of compound leaved maples and phylogenetic inference with other *Acer* species. Tree Genet. Genomes.

[25] Jiang Z.G., Wang W.H., Zhang J.B., Yang J.Y., Yang L.S., Yu H.L., Zhang Q.H. (2017). Study on rare and endangered plants in Shennongjia. Hubei Agric. Sci..

[26] Warren D.L., Glor R.E., Turelli M. (2020). ENMTools: A toolbox for comparative studies of environmental niche models. Ecography.

[27] Lin W., Ye C.J., Ming X.J., Qi J.S., Liu X., Fan G.C., Liao J.X., Wang Y.D., Liu X. (2025). Habitat suitability analysis of *Dendrobium flexicaule* using MaxEnt and InVEST models. Front. Plant Sci..

[28] Li M., Zheng C.F., Gao X.Q., Li C.H., Li Y.X., Xia X.H., Yang J., Zheng Y.Q., Huang P. (2024). Distinct ecological habits and habitat responses to future climate change in two subspecies of *Magnolia sieboldii* K. Koch, a tree endemic to East Asia. Plants.

[29] Xu H.Y., Jiang C.L., Li X., Fan H.R., Wang J.M., Li J.J. (2025). Optimized MaxEnt modeling reveals major decline and shift of giant panda habitat under CMIP6 ensemble projections. Ecol. Indic..

[30] Cobos M.E., Peterson A.T., Barve N., Osorio-Olvera L. (2019). Kuenm: An R package for detailed development of ecological niche models using Maxent. PeerJ.

[31] Chen J., Wu L., Yang C., Qiu Q., Wang Y., Li Z., Xia C. (2025). Predicting potential habitats of the endangered *Mangrove* species *Acanthus ebracteatus* under current and future climatic scenarios based on MaxEnt and OPGD models. Plants.

[32] Huang Y., Liu X., Chen T., Chen C., Luo Y., Xu L., Cao F. (2025). Potential suitable habitat range shift dynamics of the rare orchid *Cymbidium cyperifolium* in China under global warming. Plants.

[33] Jiménez-Valverde A. (2012). Insights into the area under the receiver operating characteristic curve (AUC) as a discrimination measure in species distribution modelling. Global. Ecol. Biogeogr..

[34] Phillips S.J., Anderson R.P., Schapire R.E. (2006). Maximum entropy modeling of species geographic distributions. Ecol. Model..

[35] Brown J.L., Bennett J.R., French C.M. (2017). SDMtoolbox 2.0: The next generation Python-based GIS toolkit for landscape genetic, biogeographic and species distribution model analyses. PeerJ.

[36] Songer M., Delion M., Biggs A., Huang Q. (2012). Modeling impacts of climate change on giant panda habitat. Int. J. Ecol..

[37] Gebrewahid Y., Abrehe S., Meresa E., Eyasu G., Abay K., Gebreab G., Kidanemariam K., Adissu G., Abreha G., Darcha G. (2020). Current and future predicting potential areas of *Oxytenanthera abyssinica* (*A. Richard*) using MaxEnt model under climate change in Northern Ethiopia. Ecol. Process..

[38] Xie C., Li M., Jim C.Y., Chen R.N. (2025). Distribution Pattern of Endangered *Cycas taiwaniana* Carruth. in China Under Climate-Change Scenarios Using the MaxEnt Model. Plants.

[39] Jia L.X., Sun M.M., He M.R., Yang M.F., Zhang M., Yu H. (2024). Study on the change of global ecological distribution of *Nicotiana tabacum* L. based on MaxEnt model. Front. Plant Sci..

[40] Qian H., Zhang Y., Ricklefs R.E., Wang X. (2022). Relationship of minimum winter temperature and temperature seasonality to the northern range limit and species richness of trees in North America. J. Geogr. Sci..

[41] Xu L., Myneni R., Chapin F., Callaghan T.V., Pinzon J.E., Tucker C.J., Zhu Z., Bi J., Ciais P., Tømmervik H. (2013). Temperature and vegetation seasonality diminishment over northern lands. Nat. Clim. Change.

[42] Zhou L., Ullah F., Zou J., Zeng X. (2025). Molecular and physiological responses of plants that enhance cold tolerance. Int. J. Mol. Sci..

[43] Baumgarten F., Zohner C.M., Gessler A., Vitasse Y. (2021). Chilled to be forced: The best dose to wake up buds from winter dormancy. New Phytol..

[44] Alburquerque N., García-Montiel F., Carrillo A., Burgos L. (2008). Chilling and heat requirements of sweet cherry cultivars and the relationship between altitude and the probability of satisfying the chill requirements. Environ. Exp. Bot..

[45] Tiwary R., Singh P.P., Adhikari D., Behera M.D., Barik S.K. (2024). Vulnerability assessment of *Taxus wallichiana* in the Indian Himalayan region to future climate change using species niche models and global climate models under future climate scenarios. Biodivers. Conserv..

[46] Chen W.Y., Su T. (2020). Asian monsoon shaped the pattern of woody dicotyledon richness in humid regions of China. Plant Divers..

[47] Zhai P.M., Zhang X.B., Wan H., Pan X.H. (2005). Trends in total precipitation and frequency of daily precipitation extremes over China. J. Clim..

[48] Guo H., Li D.L., Lin S., Dong Y.X., Sun L.D., Huang L.N., Lin J.J. (2013). Temporal and spatial variation of precipitation over western China during 1954–2006. J. Glaciol. Geocryol..

[49] Dai A.G. (2013). Increasing drought under global warming in observations and models. Nat. Clim. Change.

[50] Tian M., Liu Y.J., Liu Z.J., Yang T., Wang W.B., Hao B.B., Gong J.Q., Ou K.J., Su J.H. (2025). The influence of altitude and aspect on the species functional groups and diversity of plant communities in the Tangbei area of the Sanjiangyuan National Park. Grassl. Turf..

[51] Sokolova G. (2016). The influence of terrain altitude, slope exposure and slope degree on plant spatial distiribution. Acta Biol. Sibir..

[52] Gao Z.W., Ma X.Y., Tang S.Q., Han S.W., Shen Y.T., Yang X.B. (2024). Effects of terrain factors on the growth of *Platycladus orientalis* forest in Hebei Province. J. Northeast For. Univ..

[53] Lu X., Mao Q., Gilliam F.S., Luo Y., Mo J. (2014). Nitrogen deposition contributes to soil acidification in tropical ecosystems. Glob. Change Biol..

[54] Schönswetter P., Stehlik I., Holderegger R., Tribsch A. (2005). Molecular evidence for glacial refugia of mountain plants in the European Alps. Mol. Ecol..

[55] Biella P., Cornalba M., Rasmont P., Neumayer J., Mei M., Brambilla M. (2024). Climate tracking by mountain bumblebees across a century: Distribution retreats, small refugia and elevational shifts. Glob. Ecol. Conserv..

[56] Kyalo C.M., Chen L.Y., Lema M., Malombe I., Hu G.W., Wang Q.F. (2022). Multiple Pleistocene refugia and recent diversification for *Streptocarpus ionanthus* (Gesneriaceae) complex: Insights from multiple molecular sources. J. Syst. Evol..

[57] Xia X.H., Yu X.D., Wu Y.X., Liao J., Pan X.Y., Zheng Y.Q., Zhang C.H. (2025). Orogeny and high pollen flow as driving forces for high genetic diversity of endangered *Acer griseum* (Franch.) Pax endemic to China. Int. J. Mol. Sci..

[58] Xu T.Z. (1983). Geographical distribution and floristic characteristics of *Acer* in Hengduan Mountain area of China. Acta Bot. Yunn..

[59] Harris J.G.S. (1975). Tree genera—3. *Acer*—Of the maple. Arboric. J..

[60] Liu D., Yang J., Chen S., Sun W. (2022). Potential distribution of threatened maples in China under climate change: Implications for conservation. Glob. Ecol. Conserv..

[61] Hurlbert A.H., Jetz W. (2007). Species richness, hotspots, and the scale dependence of range maps in ecology and conservation. Proc. Natl. Acad. Sci. USA.

[62] Zhang J.F., Ge S.S., Li Y.T., Li J.Q. (2022). Dispersal and germination of nine maple (*Acer*, spp) trees in Changbai Mountain area. Acta Ecol. Sin..

[63] He Q.E., Shao J.H., Chen X.L., Chen L.K. (2025). Prediction and analysis of suitable habitats for *Andrographis paniculata* in China under climate change. Guangdong Agric. Sci..

[64] Wang J., Taylor A.R., D’Orangeville L. (2023). Warming-induced tree growth may help offset increasing disturbance across the Canadian boreal forest. Proc. Natl. Acad. Sci. USA.

[65] Mekonnen Z.A., Riley W.J., Randerson J.T., Grant R.F., Rogers B.M. (2019). Expansion of high-latitude deciduous forests driven by interactions between climate warming and fire. Nat. Plants.

[66] Yu F.Y., Wang T.J., Groen T.A., Skidmore A.K., Yang X.F., Ma K.P., Wu Z. (2019). Climate and land use changes will degrade the distribution of Rhododendrons in China. Sci. Total Environ..

[67] Corlett R.T., Westcott D.A. (2013). Will plant movements keep up with climate change?. Trends Ecol. Evol..

[68] De Jong P.C. (1976). Flowering and Sex Expression in *Acer* L.: A Biosystematic Study. Nederland: Mededelingen Land Bouwboge School Wageningen. Ph.D. Thesis.

[69] Sun J.W., Zheng Y.Q., Yu X.D., Xia X.H., Zhao Y.X., Wu Y.X., Zhang C.H. (2022). Floral traits and mating system of endangered species *Acer griseum*. Sci. Silvae Sin..

[70] Panetta A.M., Stanton M.L., Harte J. (2018). Climate warming drives local extinction: Evidence from observation and experimentation. Sci. Adv..

[71] Frankham R., Ballou J.D., Briscoe D.A. (2002). Introduction to Conservation Genetics.

[72] Zhao H., Zong Y.C., Zheng Y.Q. (2014). Analysis of the current situation of China’s forest genetic resources conservation. Hunan For. Sci. Technol..

[73] Chacón-Vargas K., García-Merchán V.H., Sanín M.J. (2020). From keystone species to conservation: Conservation genetics of wax palm *Ceroxylon quindiuense* in the largest wild populations of Colombia and selected neighboring ex situ plant collections. Biodivers. Conserv..

[74] Baggio-Compagnucci A., Ovando P., Hewitt R.J., Canullo R., Gimona A. (2022). Barking up the wrong tree? Can forest expansion help meet climate goals?. Environ. Sci. Policy.

