# Supplementary material for: From Glacial Refugia to Future Shifts: Unraveling the Spatiotemporal Dynamics of Endangered Acer sutchuenense Franch. Under Climate Change
Source: Biology (Basel). 2026 Feb 28;15(5):397. doi: 10.3390/biology15050397 (PMC12984191; doi:10.3390/biology15050397)
Supplement: Supplementary file 1 [file biology-15-00397-s001.zip › biology-4144333-supplementary.pdf]

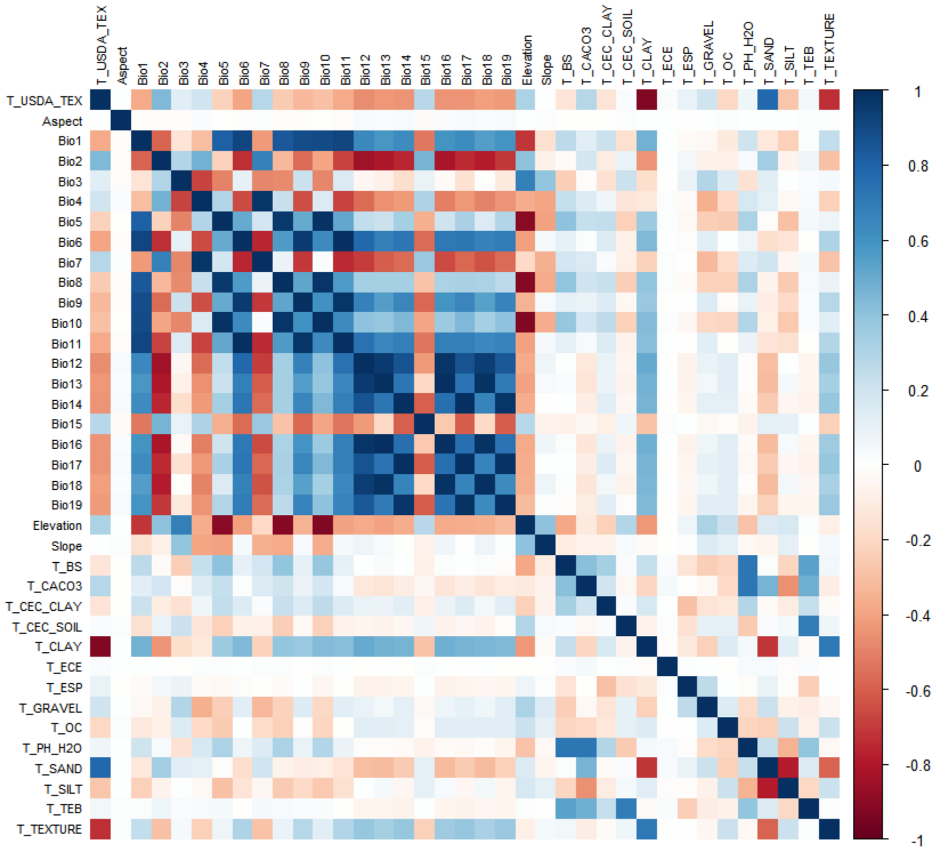

Figure S1. Correlation analysis of 37 environmental variables.

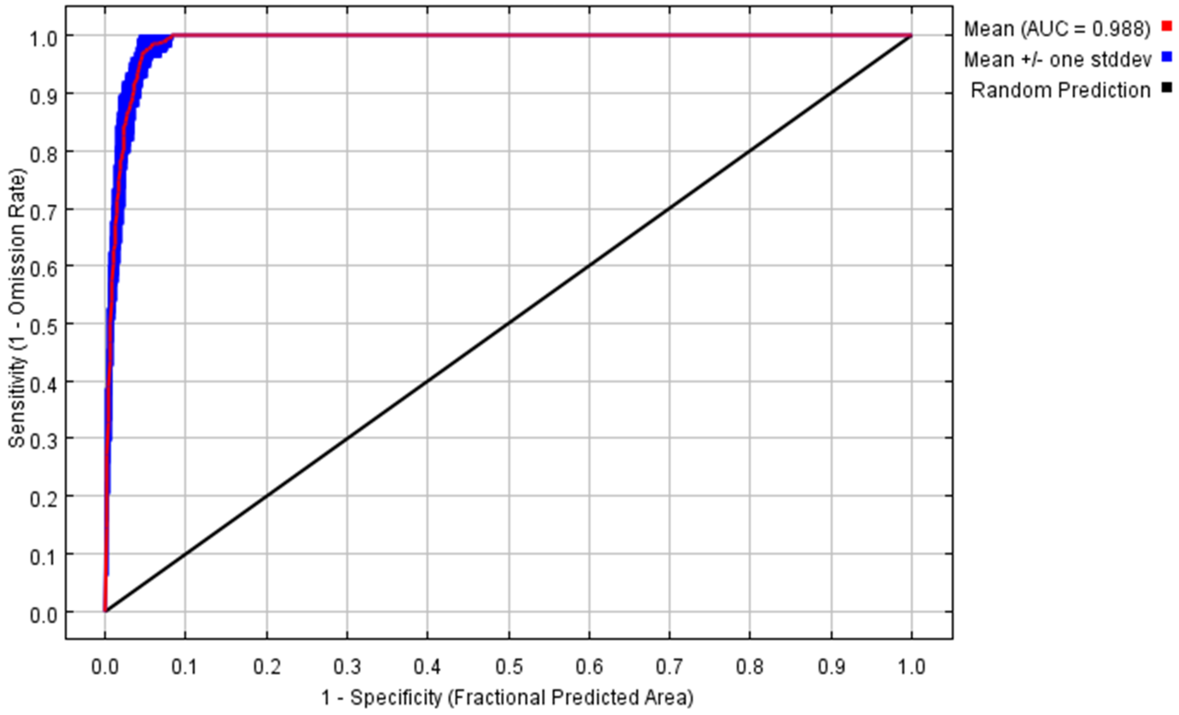

Figure S2. ROC curve simulated in the suitable habitats of *A. sutchuenense*.

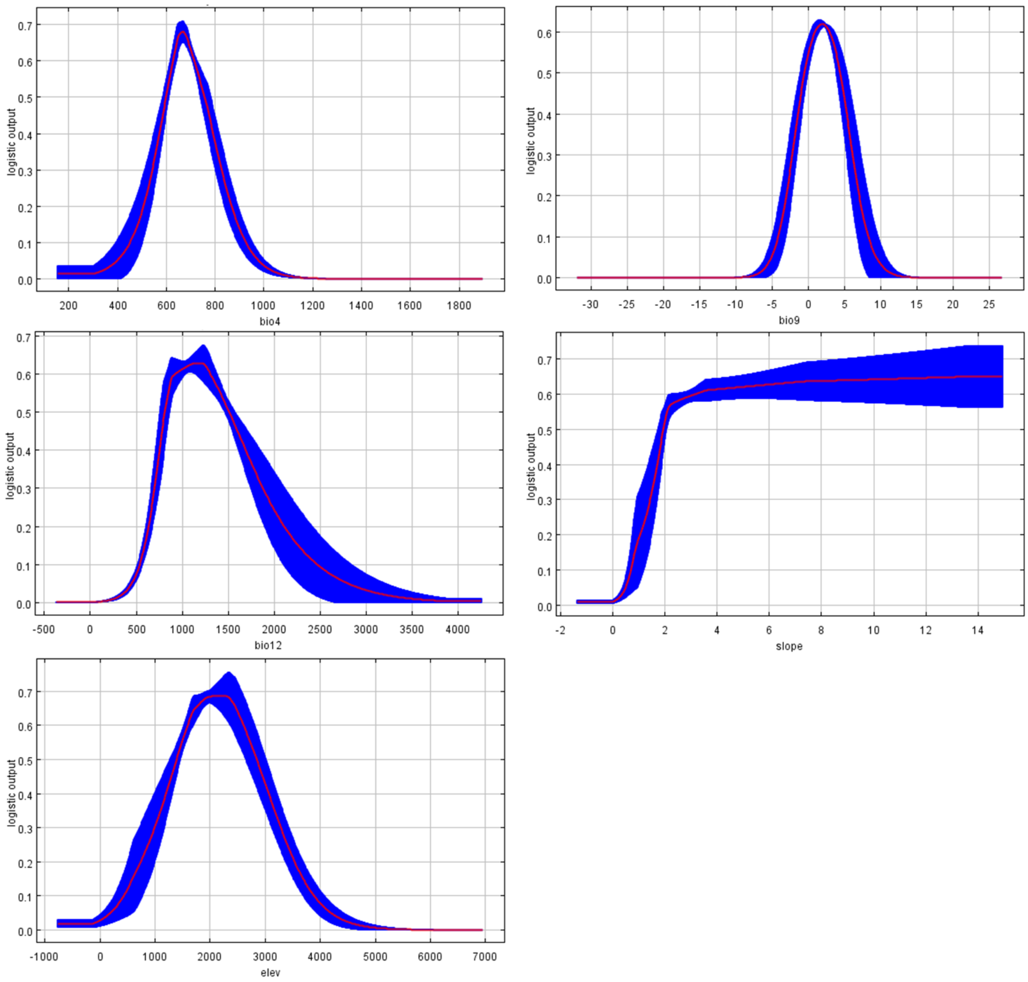

Figure S3. Response curve for five dominant climate variables.

Table S1. 37 environmental variables collected for the MaxEnt model.

Code	Environmental variables	Code	Environmental variables
Bio1	Annual mean temperature	Elev	Elevation
Bio2	Mean diurnal range	Slope	Slope
Bio3	Isothermality	Aspect	Aspect
Bio4	Temperature seasonality	T_BS	Topsoil base saturation
Bio5	Max temperature of warmest month	T_CACO_3	Topsoil calcium carbonate
Bio6	Min temperature of coldest month	T_CEC_CLAY	Topsoil CEC (clay)
Bio7	Temperature annual range	T_CEC_SOIL	Topsoil CEC (soil)
Bio8	Mean temperature of wettest quarter	T_CLAY	Topsoil clay fraction
Bio9	Mean temperature of driest quarter	T_ECE	Topsoil salinity (Elco)
Bio10	Mean temperature of warmest quarter	T_ESP	Topsoil sodicity (ESP)
Bio11	Mean temperature of coldest quarter	T_GRAVEL	Topsoil gravel content
Bio12	Annual precipitation	T_OC	Topsoil organic carbon
Bio13	Precipitation of wettest month	$T\_PH\_H_2O$	Topsoil pH (H_2O)
Bio14	Precipitation of driest month	T_SAND	Topsoil sand fraction
Bio15	Precipitation seasonality	T_SILT	Topsoil silt fraction
Bio16	Precipitation of wettest quarter	T_TEB	Topsoil TEB
Bio17	Precipitation of driest quarter	T_TEXTURE	Topsoil texture
Bio18	Precipitation of warmest quarter	T_USDA_TEX	Topsoil USDA texture classification
Bio19	Precipitation of coldest quarter		
